# Phlegmonous Gastritis With Streptococcal Toxic Shock Syndrome: A Rare Complication After Endoscopy

**DOI:** 10.7759/cureus.73972

**Published:** 2024-11-19

**Authors:** Tobias Müller, Emanuel Gmür

**Affiliations:** 1 Department of Surgery, GZO Spital Wetzikon, Wetzikon, CHE; 2 Department of Surgery, Bauchzentrum Rapperswil, Rapperswil, CHE

**Keywords:** abdominal compartment syndrome, acute abdomen, phlegmonous gastritis, toxic shock syndrome, upper endoscopy

## Abstract

Phlegmonous gastritis is a rare, suppurative bacterial infection of the gastric wall and one of the rarest complications after upper gastrointestinal endoscopy. The pathogenesis is not fully clear, but multiple risk factors have been described in literature as mucosal injury and achlorhydria.

We report a case of a 76-year-old male with vomiting and epigastric pain, two days after an upper endoscopy, who presented in septic shock. The diagnostics with computed tomography showed diffuse thickening of the gastric wall, and the differential diagnosis of phlegmonous gastritis was made. Subsequently, he developed abdominal compartment syndrome and clinically deteriorated, necessitating open partial gastric resection.

This case of a rare complication after upper gastrointestinal endoscopy with a potentially fatal outcome illustrates septic shock and abdominal compartment syndrome as severe complications. In this case, a combination of early antibiotic treatment and calculated surgical interventions showed a favorable outcome. Only a limited number of cases of phlegmonous gastritis after endoscopy have been published, and to the best of our knowledge, this is the second case of phlegmonous gastritis with subsequent abdominal compartment syndrome as a complication.

## Introduction

Phlegmonous gastritis (PG) is a rare suppurative infection of the gastric wall, and without quick diagnosis and treatment, it can be lethal. The prognosis has improved with antibiotic therapy, but, in some cases, surgery is required [1,2]. PG is located in the muscularis propria and submucosal layers in a diffuse or focal manner, mostly caused by streptococci [3]. The symptoms upper abdominal pain, nausea, and vomiting are very unspecific. The pathophysiology is not yet completely understood, but there are several risk factors (mucosal injury, alcoholism, age, immunosuppression) that have been associated with PG [2]. Nowadays, there is no consensus on the treatment of PG, which also manifests in its high mortality rates of up to 40% [2,4].

Up to today, only seven cases of PG after endoscopy have been reported [3,5-10]. All patients reported abdominal pain, nausea, and vomiting after an upper gastrointestinal (GI) endoscopy. In most cases, a total gastrectomy was necessary during the treatment [3,5,6,8-10]. We faced a complicated case with abdominal compartment syndrome (ACS) that was managed with only a partial gastrectomy. To our knowledge, this is the eighth case of post-endoscopy PG, the second case with reported acute abdominal compartment, and the first that was treated successfully with partial gastrectomy.

## Case presentation

A 76-year-old male with no pre-existing conditions or regular medication underwent an outpatient upper GI endoscopy with diagnostic biopsies as part of the workup for epigastric pain, which showed *Helicobacter pylori *gastritis. A therapy with high-dose proton pump inhibitors (PPIs), and an eradication therapy with tetracyclin, metronidazole, and bismuth were initiated.

The day after, he presented to the endoscopist with increased epigastric pain, nausea, and vomiting. Clinically, he revealed epigastric tenderness without any signs of peritonitis. After a saline infusion, the patient felt better and refused hospital admission. Two days later, he presented again to the endoscopist with persistent vomiting, tachycardia, tachydyspnea, and peritonism. He was therefore admitted to our emergency department.

**Figure 1:**
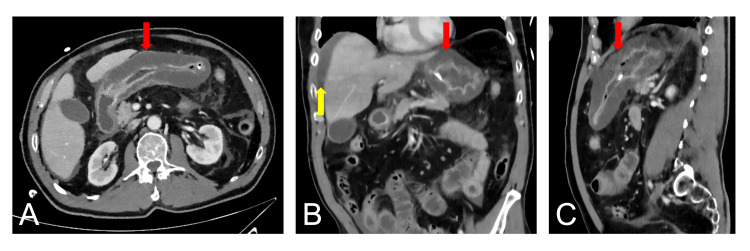
Initial imaging on admission Abdominal contrast-enhanced computed tomography scan on the day of admission revealed a circumferential thickening of the gastric wall (marked with a red arrow) and free abdominal fluid (marked with a yellow arrow). (A) Axial plane. (B) Coronal plane. (C) Sagittal plane.

At the emergency department, the patient was conscious and septic (blood pressure 80/67 mmHg, pulse 113 beats/min, temperature 37.6°C) and showed no presence of bowel sounds and mottled abdominal skin and legs. An abdominal ultrasound showed free abdominal fluid and a diffuse thickening of the gastric wall. Laboratory results on admission showed a white blood cell count of 1,400/mm^3^ (reference range: 3,500-9,600/mm^3^), C-reactive protein of 448 mg/L (reference range: < 5 mg/L), and lactate of 7.9 mmol/L (reference range 1-2.2 mmol/L). Table 1 shows a summary of the laboratory results on admission. Because of the sepsis, an empiric antibiotic therapy with piperacillin/tazobactam was started, and the diagnosis was completed with an abdominal computed tomography (CT) that showed a diffuse thickened gastric wall with no signs of perforation (Figure 1). The suspected diagnosis of a septic PG was made, a non-surgical treatment with empiric antibiotics was chosen, and the patient was transferred to the intensive care unit (ICU).

**Table 1:** Laboratory results on admission

Blood Work	Patient Test Result	Reference Range
Hemoglobin	177 g/L	135-175 g/L
Hematocrit	51%	42-53%
Wight blood cell count	1,400/mm^3^	3.5-9.6/mm^3^
C-reactive protein	448 mg/L	<5 mg/L
Lactate	7.9 mmol/L	1-2.2 mmol/L
Creatinine	276 µmol/L	62-106 µmol/L

**Figure 2:**
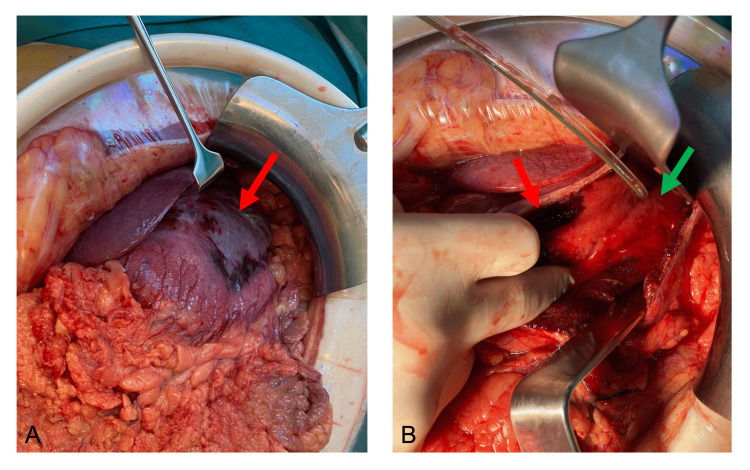
Intraoperative images Intraoperative pictures of the emergency laparotomy. (A) Intraoperative situs after laparotomy with partial extended gastric necrosis (marked with a red arrow). (B) Intraoperative situs after gastrotomy that shows the vital gastric mucosa (marked with a green arrow) and the necrotic mucosa (marked with a red arrow).

During the night, the patient’s need for vasopressors and bladder pressure increased steadily. With the diagnosis of an acute ACS (intra-abdominal pressure [IAP] of 30 mmHg [reference range: 0-5 mmHg]), an explorative laparotomy was performed. During the laparotomy, there was a lot of clear ascites and some sharply demarked necrosis on the ventral part of the gastric corpus. For further inspection of the mucosa, a gastrotomy was performed, which showed vital mucosa (Figure 2). The necrosis was locally debrided, and the stomach closed longitudinally with interrupted stitches (2-0 PDS) after partial gastrectomy of the anterior gastric wall. A vacuum-assisted closure (VAC) dressing was used to close the abdomen, and the patient was transferred back to the ICU.

**Figure 3:**
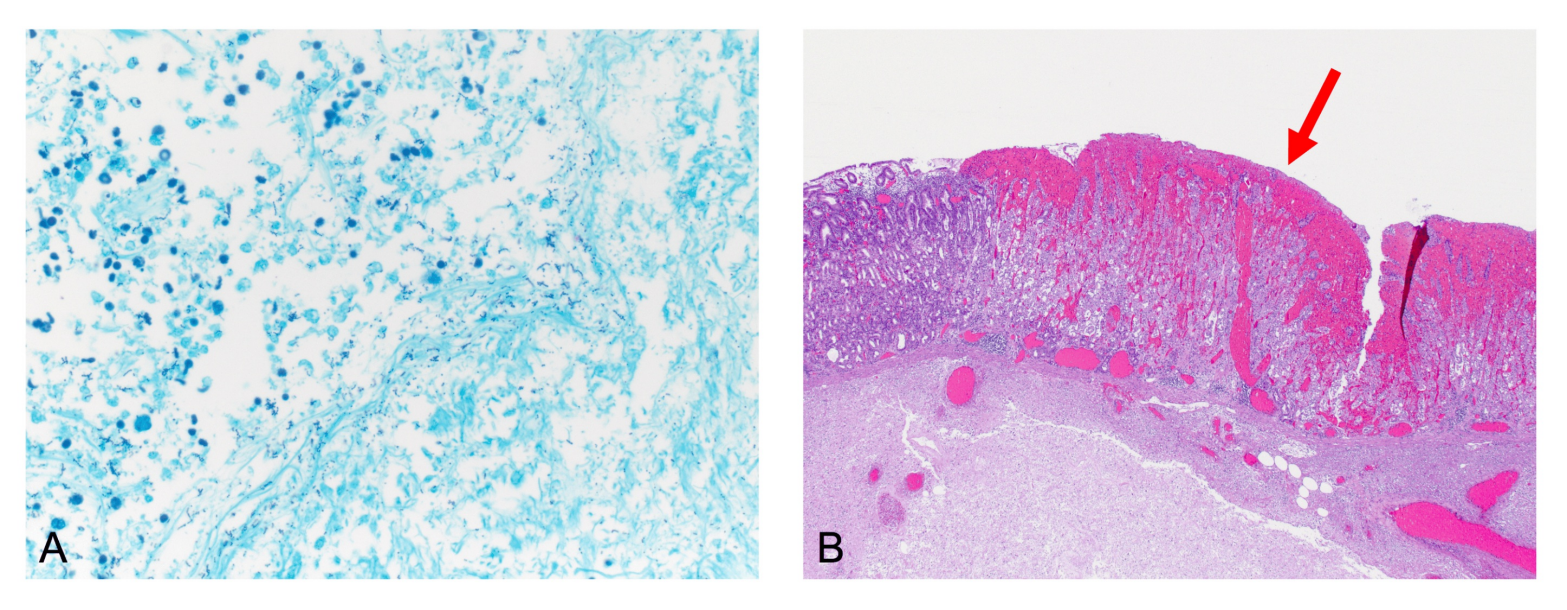
Histology (A) Several cocci bacteria (dark dots) in the gastric mucosa (Giemsa staining; 400x). (B) B transition of the vital mucosa (on the left side) to the necrotic mucosa (on the right side, marked with a red arrow) (hematoxylin and eosin stain; 25x).

Tissue samples were sent for cultivation, which showed the growth of *Streptococcus pyogenes* and *Candida albicans*. The histological sample showed extended wall necrosis and plenty of intramurally located cocci bacteria (Figure 3). Based on the findings, the diagnosis of a streptococcal toxic shock syndrome and PG with abdominal compartment syndrome was established.

After the first surgery, the patient developed multi-organ failure with anuria with the need for hemodialysis and prolonged mechanical ventilation. On the fourth day after initial presentation, a second look was performed under stabilized conditions, which showed no further findings, and the abdomen was again closed with a VAC dressing, and an insertion of a nasoduodenal feeding tube for early post-pyloric feeding was performed. On the fifth day, there was blood in the VAC container, and thus the patient was rushed to surgery, which showed a perforated subcapsular liver hematoma of segment III that was then coagulated. Further looks were performed on the 8th, 11th, and 13th days after the initial presentation, during which a splenic abscess was drained and parts of the perihepatic hematoma were removed. Meanwhile, a tracheotomy was performed, and the antimicrobial therapy was expanded with fluconazole for 18 days after multiple intra-abdominal samples showed the growth of *Candida albicans*. On the 15th day after presentation, the abdomen was closed after leaving two drainages (perisplenic and between the liver and stomach). The antibiotic treatment with piperacillin/tazobactam was stopped after 17 days. Due to persistent anuria, hemodialysis was continued.

After tracheotomy, he subsequently developed an aspiration pneumonia with the need of vasopressors and antibiotic therapy with cefepime after *Escherichia coli *was isolated in the tracheal fluid. After that, further recovery was set back due to several infectious complications and a severe critical illness polyneuropathy. Follow-up gastroscopy revealed a vulnerable gastric mucosa with subtotal stenosis with visible suture material. After 62 days, the patient was able to be decannulated, progressed in his recovery, and was transferred to the surgical ward after 88 days.

After 108 days, the patient was discharged and transferred to rehabilitation. He still needed hemodialysis three times a week but was able to eat soft food again.

## Discussion

PG is a rare suppurative bacterial infection of the stomach and a very rare complication after GI endoscopy, with a potentially fatal outcome. The first description was in the 18th century by Cruveilhier, and since then, approximately 450-500 cases have been reported [2-4,11,12]. The mortality pre-antibiotic era was up to 92%, and the reported mortality up to 2003 was up to 42% [2]. Up to today, only seven cases of PG after upper GI endoscopy have been reported [3,5-10], with only one case with ACS as a complication [3].

The clinical presentation of PG is non-specific and includes upper abdominal pain, nausea, vomiting, fever, chills and hematemesis. Of these, abdominal pain and vomitus were reported as the most common symptoms [2,4]. In our case, the patient presented with the symptoms of increased epigastric pain, nausea, and vomitus.

Several possible risk factors such as alcoholism, mucosal injury, achlorhydria, advanced age, and immunosuppression have been suggested; however, the exact pathogenesis of PG is not known [2,3]. The literature suspects different routes of infection, such as direct and hematogenous infection. Moreover, a localized form and a diffuse form of PG have been reported. Out of the two forms, the diffuse form showed a significantly higher mortality rate (10% vs 54%) [2].

In our case, the present risk factors can be assumed to be advanced age, mucosal lesions caused by the performed biopsies, and possible ulcerations due to the Helicobacter pylori infection with high-dose PPI treatment.

The diagnostic work-up for PG includes clinical examination, blood work, imaging (abdominal ultrasound, CT scan), and upper GI endoscopy with possible endoscopic ultrasound. Typical findings are elevated inflammation values, and thickening and edema of the gastric wall [2-4,13]. Our case showed all these aforementioned findings, even though an endoscopy (said to be the gold standard in some studies) was not performed in this case due to the cardiopulmonary instability of the patient. When finding a diffuse thickened gastric wall, including other differential diagnoses such as gastric carcinoma, MALT lymphoma, leiomyoma, and GI stromal tumors is necessary. Typical indicators for PG is fibrinopurulent exudate with an edematous, hyperemic mucosa in the upper GI endoscopy, and a hypoechoic submucosa in the endoscopy ultrasound [2,4].

*Streptococcus pyogenes* is the most reported pathogen for PG (up to 70%) [2,12,14], as it was in our case. Other common pathogens are *Enterobacter* spp., *Escherichia coli*, and *Proteus* spp., and these often occur as polymicrobial infections. Infections with *Streptococcus *ssp. have a higher mortality rate of 53% and a poor prognosis [2]. The higher mortality is suspected due to the streptococcal toxic shock syndrome, which is caused by superantigens produced by the *Streptococcus* spp. [11,13,15].

The treatment of PG can be surgical or conservative, as there are reported cases with successful conservative therapy. However, a combination of broad-spectrum antibiotics and early gastric resection showed the best patient survival rates [2,4,11]. Surgery is mandatory if local complications such as perforation, gastric necrosis, or, as in our case, ACS are present. If the patient is unresponsive to conservative treatment, surgical treatment has been associated with lower mortality rates (20% with surgery vs. 50% with medical treatment) [2]. Most commonly, a total gastrectomy was performed, but some cases with partial gastrectomy were reported [2,4]; however, most of these patients passed away or subsequently underwent total gastrectomy. To the best of our knowledge, none of the aforementioned cases with partial gastrectomy were post-endoscopy PG, which differs from our case.

Our patient developed an ACS, due to which an explorative laparotomy and partial gastrectomy were performed. The ACS is an often-overlooked medical condition in medical ICUs and is the result of large fluid resuscitation, acidosis, sepsis, and presence of ascites with subsequent multi-organ failure. If overlooked, rapid deterioration is often the case [16]. ICU patients are at a higher risk for intra-abdominal hypertension (IAH) due to the underlying conditions. IAH is defined as an increased IAP > 12 mmHg, and ACS is a combination of an IAP over 20 mmHg and new organ dysfunction [17,18]. The IAP in our patient was 30 mmHg with progressive deterioration, which lead to the diagnosis of ACS and was treated according to the WSES (World Society of Emergency Surgery) guidelines [18]. In this case, early broad-spectrum antibiotics and rapid surgical decompression of the ACS with a prolonged ICU treatment led to a favorable outcome for the patient.

As far as we know, this is the eighth reported case of PG after upper GI endoscopy with biopsy, and the second reported case with ACS as a complication (Table 2) [3,5-10]. PG is a very rare bacterial infection of the stomach and an even rarer complication after upper GI endoscopy. Because of the rarity of PG, the awareness of its possibility in patients after upper GI endoscopy with symptoms is important, and an early diagnosis and treatment should be maintained. In the early stage and focal disease, the patient can be managed effectively with broad-spectrum antibiotics. In the later stage with diffuse disease and complications, such as perforation or ACS, surgical management is still the most favorable way to go.

**Table 2:** Summary of case reports of acute phlegmonous gastritis after upper GI endoscopy and biopsy

Author	Year of Publication	Country	Age	Sex	Intervention	Abdominal Compartment	Surgery	Outcome
Modares and Tabari [3]	2021	Canada	67	Male	Upper GI endoscopy with biopsy	Yes	Total gastrectomy	Discharged
Sahnan et al. [5]	2013	UK	56	Female	Argon plasma coagulation of gastric antral vascular ectasia	No	Total gastrectomy	Death
Ajibe et al. [6]	2008	Japan	74	Male	Endoscopic submucosal dissection	No	Total gastrectomy	Discharged
Itonaga et al. [7]	2012	Japan	70	Female	Endoscopic ultrasound-guided fine-needle aspiration	No	None	Discharged
Lee et al. [8]	2005	Korea	68	Female	Endoscopic mucosal resection	No	Total gastrectomy	Discharged
Lifton and Schlossberg [9]	1982	United States	70	Female	Endoscopic polypectomy	No	Total gastrectomy	Discharged
Shirai et al. [10]	2003	Japan	68	Male	Upper GI endoscopy with biopsy	No	Total gastrectomy	Discharged

## Conclusions

PG is a rare suppurative bacterial infection of the stomach and one of the rarest complications after upper GI endoscopy. The awareness of the possibility for PG after endoscopy and the initiation of an early treatment could improve its prognosis.

The outcome of our case could lead to the conclusion that if the condition of the patient allows it, a partial gastrectomy or local necrosectomy could be enough to treat a PG, and total gastrectomy (and its potential consequences) is not always necessary. However, it is too early for a recommendation, and further research is necessary.
